# Understanding Pathways into Care homes using Data (UnPiCD study): a retrospective cohort study using national linked health and social care data

**DOI:** 10.1093/ageing/afac304

**Published:** 2022-12-29

**Authors:** Jennifer Kirsty Burton, Giorgio Ciminata, Ellen Lynch, Susan D Shenkin, Claudia Geue, Terence J Quinn

**Affiliations:** Academic Geriatric Medicine, School of Cardiovascular & Metabolic Health, University of Glasgow, Glasgow, Scotland; Health Economics and Health Technology Assessment, School of Health & Wellbeing, University of Glasgow, Glasgow, Scotland; Social Care Analytical Unit, Health and Social Care Analysis, Scottish Government, Edinburgh, Scotland; Ageing and Health Research Group and Advanced Care Research Centre, Usher Institute, University of Edinburgh, Edinburgh, Scotland; Health Economics and Health Technology Assessment, School of Health & Wellbeing, University of Glasgow, Glasgow, Scotland; Academic Geriatric Medicine, School of Cardiovascular & Metabolic Health, University of Glasgow, Glasgow, Scotland

**Keywords:** Care home, hospital, routine data, data linkage, social care, older people

## Abstract

**Background:**

Pathways into care are poorly understood but important life events for individuals and their families. UK policy is to avoid moving-in to care homes from acute hospital settings. This assumes that moves from secondary care represent a system failure. However, those moving to care homes from community and hospital settings may be fundamentally different groups, each requiring differing care approaches.

**Objective:**

To characterise individuals who move-in to a care home from hospital and compare with those moving-in from the community.

**Design and setting:**

A retrospective cohort study using cross-sectoral data linkage of care home data.

**Methods:**

We included adults moving-in to care homes between 1/4/13 and 31/3/16, recorded in the Scottish Care Home Census. Care home data were linked to general and psychiatric hospital admissions, community prescribing and mortality records to ascertain comorbidities, significant diagnoses, hospital resource use, polypharmacy and frailty. Multivariate logistic regression identified predictors of moving-in from hospital compared to from community.

**Results:**

We included 23,892 individuals moving-in to a care home, 13,564 (56.8%) from hospital and 10,328 (43.2%) from the community. High frailty risk adjusted Odds Ratio (aOR) 5.11 (95% Confidence Interval (CI): 4.60–5.68), hospital discharge with diagnosis of fracture aOR 3.91 (95%CI: 3.41–4.47) or stroke aOR 8.42 (95%CI: 6.90–10.29) were associated with moving-in from hospital. Discharge from in-patient psychiatry was also a highly significant predictor aOR 19.12 (95%CI: 16.26–22.48).

**Conclusions:**

Individuals moving-in to care homes directly from hospital are clinically distinct from those from the community. Linkage of cross-sectoral data can allow exploration of pathways into care at scale.

## Key Points

- Pathways into care home are a topic of public, professional and policy interest.
- Individuals moving-in to care homes from hospital are clinically distinct from those moving-in from the community.
- Differences include greater dependency, frailty and recent health events including fracture, stroke, and significant mental illness.
- National cross-sectoral data linkage between social care and health data is feasible as a research methodology.
- However, linked data are biased towards health measures with limited information on care needs, complexity and social networks.

## Background

When used appropriately, moving-in to a care home makes a positive contribution to the care landscape, offering best care to individuals who require this level of support and reducing demand on healthcare systems [1, 2]. However, use of care home placement—who moves-in to a care home, by what route and for what purpose—is a source of unexplained variation across the UK and internationally [3–6].

Longitudinal predictors of requiring care home admission for community dwelling older people include older age, cognitive and functional impairment, requirement for support in activities of daily living, living alone, not owning your home low self-rated health and polypharmacy [7, 8]. Among hospitalised adults older age, female sex, dementia and functional dependency were all associated with increased likelihood of moving-in to a care home at discharge [9]. These studies have considered moving-in to a care home compared to remaining in your own home, without comparing and contrasting the route by which individuals move-in.

Moving-in to a care home is a complex decision for individuals, their families and professionals [10–12]. The recognition that the decision should be avoided during a time of crisis [13, 14] has informed policy in the UK advocating against moving-in to a care home from an acute hospital admission [15, 16]. However, this remains a common pathway without established standards to support it [17]. Importantly, it is not known if those moving-in to care homes directly from a hospital admission are similar or different to those moving-in from the community. Understanding who requires care in a care home setting is critical to inform effective care service planning, and to reconfigure pathways to support individuals moving-in to care.

The Scottish Care Home Census (SCHC) is a unique data resource, with data collected on those living in care homes in Scotland, irrespective of the sector providing care (e.g. private, voluntary or local authority) or their source of funding [18]. Data linkage offers the potential to enhance the information available in single data sources and explore complexity in everyday practice [19]. Using a linked dataset combining social care generated data (from SCHC) with routinely collected health data, offered the opportunity to look at pathways into care homes across Scotland. Our aim was to characterise individuals who move-in to a care home from hospital and those moving-in from the community, identifying factors associated with moving-in from hospital. We wanted to explore if there were differences between those experiencing the two pathways moving-in to care homes.

## Methods

### Study design

We performed a retrospective observational cohort study of adults moving-in to care homes in Scotland between 1 April 2013 and 31 March 2016, reported as recommended in the RECORD guidance [20].

### Setting and care context

Care homes in Scotland are defined as: 24-hour residential care facilities for adults, some of which have on-site registered nursing staff, all of whom are registered by the Care Inspectorate [21]. This include services for older adults (74% of homes), those with learning disabilities (16.5%), mental health problems (5%), physical disabilities (3%) and substance misuse needs (1.5%) including those living with the chronic consequences of alcohol dependency [22]. Staffing data are not published nationally at care home level. We do not have comprehensive national data on the complexity and dependency of care home residents in Scotland.

Care homes can provide short-stay or long-stay care, defined by the Care Inspectorate as those whose length of stay is anticipated to be six weeks or longer. Average length of stay among older adults in Scotland’s care homes is 520 days [23]. No resident-level data are collected within the SCHC on short stays, such as respite care, which can be offered in standalone specialist respite homes, or within other care home services. During the study period, use of care homes for intermediate care (as a step-down from hospital) was limited. Although there are no national data collected in Scotland to formally quantify this, unlike similar national audit data available in England.

Scotland has financial allowances towards the costs of personal care and nursing care, which can be used by care home residents to contribute to the costs of their care provided they are assessed as eligible by their Local Authority [24]. There are means-tested assessments to determine eligibility for state funding towards care home placement, other individuals fund their own care without state support.

### Indexing and linkage method

We used the Scottish Care Home Census (SCHC) to define the eligible population for inclusion. This relied on linkage between the SCHC and other national data sources, to enrich the available information, using the Community Health Index (CHI) number as the identifier for linkage. Details are included in Supplementary materials 1. In summary, not all records were sufficiently complete in terms of available identifiers to enable them to be indexed to CHI [25]. Records in the SCHC that could not be indexed to CHI were excluded.

### Data sources

#### SCHC

Annual data collection from care homes in Scotland, describing the activity of the care home over the preceding financial year (1 April–31 March) include aggregate-level data on the service and individual-level data about long-stay residents (defined as those whose length of stay is anticipated to be six weeks or longer). Financial years 2013/14, 2014/15 and 2015/16 were used, based on completeness of data enabling linkage to other data sources.

#### Scottish morbidity records 01, 04 & 50

General acute inpatients and day case records; mental health inpatients and day case records and geriatric long-stay records from Scottish hospitals; extract of admissions from 1 April 2010 to 31 March 2016.

#### Prescribing information system

National community prescribing dataset based on dispensing of prescribed medicines; extract from 1 April 2010 to 31 March 2016.

#### National Records of Scotland mortality records

Mortality records of deaths registered in Scotland from 01 April 2010 to 31 May 2020 (follow-up period extended from 31 March 2016 to enable further analysis).

### Participants

Only adults (aged ≥18 years when moving-in) were included. Only an individual’s first move into a care home within the dataset was included. Duplicate records reported in multiple years of the SCHC if the individual was still resident in the home were removed.

The SCHC source of admission variable includes six options (another care home; hospital; own home; sheltered housing; supported accommodation; other/not known). The cohort needed to be divided into three distinct groups for our work (hospital; community encompassing own home, sheltered housing and supported accommodation and transfer from another care home).

For individuals with Scottish Morbidity Records (SMR) records in the study period, the following definitions were used.

### Moving-in to care home

- from hospital: evidence of hospital discharge within ten days of care home admission (defined as from three days before date of care home admission to seven days after date of care home admission)
- from the community: no evidence of hospital discharge within ten days of care home admission (as above)
- from another care home: transferred from another care home and no evidence of hospital discharge within ten days of care home admission (excluded from study)

We also compared care home date of admission against National Records of Scotland deaths to verify individuals’ status at study onset. Where issues arose around overlapping dates, missing dates and non-hospital locations, rules were created for manual review to maximise inclusion (Supplementary materials 2-3).

If an individual had no records in the hospital admission datasets (SMR01/04/50), they were classified as moving-in from the community, or moving-in from another care home if indicated by the SCHC source of admission variable.

### Hospital episode linkage

In SMR01/04/50 hospital admissions are composed of episodes of care (based on moving ward/department/hospital) and each episode of an inpatient stay has a location code. The episode data were linked into a single complete admission and the last location was checked against the institution code lookup [26] to confirm this was an NHS hospital or hospice and sub-categorise hospital types (Supplementary materials 4).

### Variables for analysis

**SCHC:** Age at care home entry, sex, ethnicity, funding, personal/nursing care allowance, receiving nursing care within the care home, diagnoses—contemporary variable definitions available [25].

**SMR01/50:** prior hospital admissions, comorbidities (Supplementary materials 5), location.

**SMR04:** prior hospital admissions, psychiatric comorbidities (Supplementary materials 5), location.

**Prescribing information system:** dispensed medications (Y/N), frequency of prescriptions (days), mean number and range of dispensed items per month.

**Derived:** comorbidity (Charlson Index); [27, 28] frailty (Hospital Frailty Risk Score); [29] hospital admission with any fracture, cancer, dementia, delirium or stroke in six months before moving-in to care home (Supplementary materials 5). These conditions were selected as those known to be associated with need for care, common in those living in care homes or life changing in terms of their impact on function/dependency [30–36].

### Statistical methods

A logistic model was used to examine predictors associated with moving-in to a care home from hospital both unadjusted and adjusted. The following variables and diagnoses were included in the adjusted model (age, sex, funding, receiving nursing care in the home, dementia, hospital discharge diagnoses in three years and six months before moving-in to care home, hospital admissions, prescribed medications and frailty). These were identified *a priori* testing clinical hypotheses of factors potentially associated with care home pathways. Analyses were conducted in Stata (16.1, StataCorp LLC, College Station, TX).

### Sensitivity analysis

A pre-planned sensitivity analysis was undertaken in which individuals were classified as moving-in to a care home from hospital only when the SCHC date of moving-in to the care home and date of hospital discharge matched exactly.

### Permissions and governance

Ethical approval was obtained from South Central—Hampshire B Research Ethics Committee (16/SC/0242). Permission for linking national data was granted by the Public Benefit and Privacy Panel for Scotland (1516–0438) and Scottish Government Social Care Analysis Division.

All data were provided by the electronic data research and innovation service (eDRIS). Anonymised data without personal identifiers were provided to the research team in the secure National Safe Haven, with remote researcher access. All outputs were subject to information governance and disclosure control by eDRIS.

## Results

### Cohort definition

**Figure 1:**
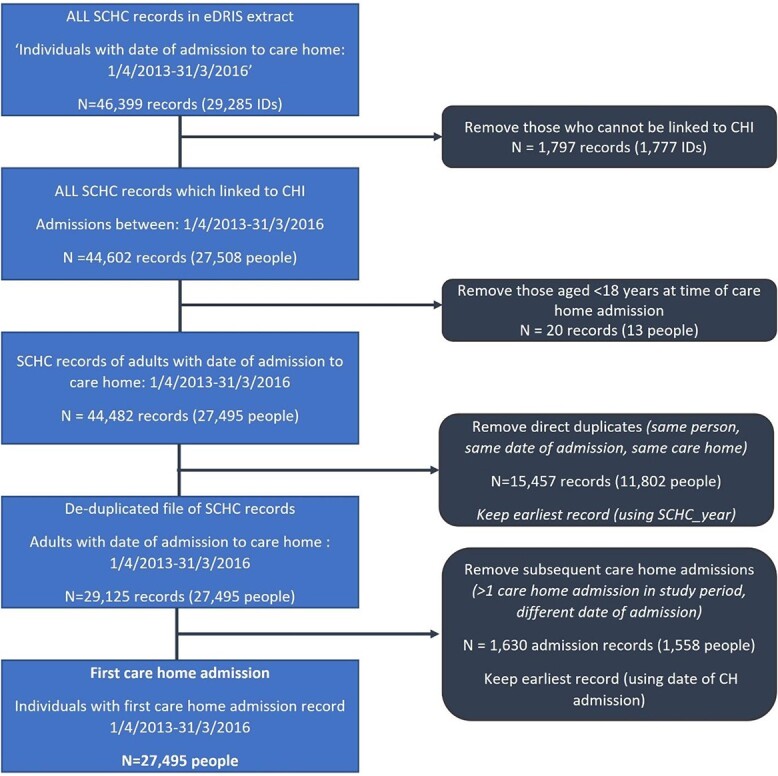
Defining Cohort for Analysis from Scottish Care Home Census Data. Footnotes: Records—individual entries from the SCHC data. IDs—identification numbers on the submitted SCHC entries. People—records belonging to the same individual, defined using the CHI as individual identifier.

From 46,399 records in the SCHC, 44,602 records representing 27,508 people could be indexed to CHI (Figure 1). After applying eligibility criteria and de-duplication 27,495 individuals were included. Of these, 24,994 (90.9%) had any records of hospital admissions between 1 April 2010 and 31 March 2016.

The analysis cohort was defined using the linked dataset. In total, 1,792 records (7.2% of those with SMR records) underwent manual review. Following this process, 252 records (1.0% of those with SMR records) were removed as there was no way to reconcile the dates and data presented. A median of one (range one to three) record(s) per care home were excluded.

We identified 3,351 individuals moving-in from another care home who were excluded. The final cohort for analysis included 23,892 individuals moving-in to a care home between 1 April 2013 and 31 March 2016, 13,564 (56.8%) moved-in from hospital and 10,328 (43.2%) moved-in from the community (Table 1).

**Table 1:** Comparing data sources in allocating source of admission

Defining data source	Source of care home admission			
	Hospital *N* (%)	Community^a^ *N* (%)	Another care home *N* (%)	Other/not known^b^ *N* (%)
**Scottish Care Home Census allocation**	12,320 (45.2)	9,874 (36.2)	3,430 (12.6)	1,619 (5.9)
**Scottish Care Home Census alone** (individuals with no hospital records in study period) **(*n* = 2,492)**^**c**^	-	1,961 (7.2)	531 (1.9)	-
**Scottish Care Home Census and Scottish Morbidity Records** (individuals with hospital records in study period) **(*n* = 24,751)**^**c**^	13,564 (49.8)	8,367 (30.7)	2,820 (10.4)	-
**Full cohort of care home admissions** ***N* = 27,243** ^**d**^	13,564 (49.8)	10,328 (37.9)	3,351 (12.3)	-
**Analysis cohort of care home admissions** ***N* = 23,892** ^**e**^	13,564 (56.8)	10,328 (43.2)	-	-

^a^Includes own home, sheltered accommodation and supported accommodation

^b^Includes blank, other or not known

^c^Excludes *n* = 252 cases removed as described earlier (9 from no hospital records group, 243 from hospital records group)

^d^Including all those with records in the SCHC alone (classified based on SCHC information) and those with linked SMR records used to inform their classification.

^e^Excludes those classified as moving-in from another care home.

### Hospital classification

In those moving-in to the care home from hospital, the type of hospital is classified, based on their last hospital location (Table 2). Rehabilitation or community hospitals account for 57.7% of discharge locations, with 33.3% discharged directly from an acute hospital and 7.1% from psychiatry. The median cumulative time spent in hospital in the six months before moving-in to the care home from hospital is 84 days, with the shortest stays among those moving-in from acute hospitals (60 days) and the longest in those moving-in from psychiatry (139 days).

**Table 2:** Classification of hospital type and median cumulative length of hospital stay for those moving-in to a care home from hospital

	Number (%)	Median cumulative length of hospital stay in six months before moving-in to care home from hospital [IQR]
Acute hospital	4,523 (33.3)	60 days [33–100]
Acute hospital (Island location)	162 (1.2)	70 days [39–136]
Hospice or NHS Hospital-Based Complex Clinical Care Unit	85 (0.6)	84 days [53–161]
Psychiatric hospital	966 (7.1)	139 days [79–184]
Rehabilitation & Community hospitals	7,828 (57.7)	93 days [62–137]
**Total population moving to care home from hospital**	**13,564**	84 days [51–131]

IQR—interquartile range

Hospital type based on SMR01/04/50 last location of discharge

### Characteristics of those moving-in from hospital compared to those moving-in from the community

Table 3 includes an abbreviated description of the population, comparing those experiencing the two pathways into care homes (full description in Supplementary Table 1). Dementia (from the SCHC or hospital discharge diagnosis) was common, affecting ~60% of those moving-in to a care home. The ICD-10 codes for delirium superimposed on dementia and confusion were not identified in the dataset. The proportion of the cohort coded as experiencing common conditions among older adults was often low, e.g. depression (3.6%), incontinence (3.8%). Polypharmacy was evident in 60% of those moving-in to care homes.

**Table 3:** Abbreviated cohort description of those moving-in to care homes from hospital and from the community

	Whole analysis cohort *N* = 23,892 people (%)	Moving-in from hospital *N* = 13,564 people (%)	Moving-in from community *N* = 10,328 people (%)
**Age band moving-in to care home** <60 years 60–69 70–79 80–89 90–99 >100 years	1,168 (4.9) 1,101 (4.6) 4,274 (17.9) 11,087 (46.4) 6,029 (25.2) 233 (1.0)	475 (3.5) 679 (5.0) 2,468 (18.2) 6,413 (47.3) 3,404 (25.1) 125 (0.9)	693 (6.7) 422 (4.1) 1,806 (17.5) 4,674 (45.3) 2,625 (25.4) 108 (1.0)
Female sex	15,593 (65.3)	8,568 (63.2)	7,025 (68.0)
White Ethnic Group	23,003 (96.3)	13,071 (96.4)	9.932 (96.2)
Mainly Local Authority funding Mainly NHS funding Mainly Private funding	15,670 (65.6) 612 (2.6) 7,588 (31.8)	8,729 (64.3) 552 (4.1) 4,276 (31.5)	6,941 (67.2) 60 (0.6) 3,312 (32.1)
Not receiving nursing care once in care home Receiving nursing care once in care home	8,342 (34.9) 15,532 (65.0)	3,654 (26.9) 9,900 (73.0)	4,688 (45.4) 5,632 (54.5)
**Conditions in Scottish Care Home Census** Dementia diagnosed Dementia (not medically diagnosed) Hearing impairment Learning disability Mental health problems excluding dementia Other physical disability or chronic illness Visual impairment	12,274 (51.4) 1,918 (8.0) 2,512 (10.5) 505 (2.1) 1,443 (6.0) 10,402 (43.5) 3,743 (15.7)	6,715 (49.5) 1,078 (8.0) 1,413 (10.4) 165 (1.2) 907 (6.7) 6,583 (48.5) 2,089 (15.4)	5,559 (53.8) 840 (8.1) 1,099 (10.6) 340 (3.3) 536 (5.2) 3,819 (37.0) 1,654 (16.0)
**Inpatient hospital diagnoses in three years before moving-in to care home** Any fracture Cancer Chronic cardiovascular disease (including heart failure) Chronic kidney disease Chronic liver disease Chronic respiratory disease Delirium Delirium superimposed on dementia Dementia Depression **Inpatient hospital diagnoses in three years before moving-in to care home (cont)** Diabetes Falls Hip fracture Incontinence Neurodegenerative conditions (including Parkinson’s disease) Stroke Syncope and collapse	4,441 (18.6) 1,774 (7.4) 7,763 (32.5) 3,147 (13.2) 204 (0.9) 3,355 (14.0) 2,970 (12.4) 0 7,700 (32.2) 859 (3.6) 3,062 (12.8) 6,570 (27.5) 2,431 (10.2) 917 (3.8) 1,475 (6.2) 1,998 (8.4) 2,028 (8.5)	3,238 (23.9) 1,312 (9.7) 5,423 (40.0) 2,245 (16.6) 143 (1.1) 2,376 (17.5) 2,235 (16.5) 0 5,319 (39.2) 621 (4.6) 2,147 (15.8) 4,648 (34.3) 1,819 (13.4) 689 (5.1) 1,033 (7.6) 1,592 (11.7) 1,356 (10.0)	1,203 (11.6) 462 (4.5) 2,340 (22.7) 902 (8.7) 61 (0.6) 979 (9.5) 735 (7.1) 0 2,381 (23.1) 238 (2.3) 915 (8.9) 1,922 (18.6) 612 (5.9) 228 (2.2) 442 (4.3) 406 (3.9) 672 (6.5)
**Hospital use and significant events in six months before moving-in to care home** Mean number of hospital admissions per person [SD] Median cumulative length of hospital stay per person [IQR] Hospital discharge from in-patient psychiatry Hospital discharge with diagnosis of cancer Hospital discharge with diagnosis of dementia Hospital discharge with diagnosis of delirium Hospital discharge with diagnosis of fracture Hospital discharge with diagnosis of stroke	1.2 admissions [1.15] 70 days [34–116] 2,239 (9.4) 1,247 (5.2) 6,740 (28.2) 2,035 (8.5) 2,545 (10.7) 1,260 (9.4)	1.7 admissions [1.06] 84 days [51–131] 2,032 (15.0) 1,020 (7.5) 5,436 (40.1) 1,656 (12.2) 2,157 (15.9) 1,133 (8.4)	0.6 admissions [0.92] 21 days [8–50] 207 (2.0) 227 (2.2) 1,304 (12.6) 379 (3.7) 388 (3.8) 127 (1.2)
**Community medication use in three years before moving-in to care home** Mean dispensed items/month [SD] Range dispensed items per month	6.5 items [5.18] 0 to 84 items	6.6 items [5.24] 0 to 84 items	6.3 items [5.10] 0 to 78 items
**Hospital Frailty Risk Score before moving-in to care home** Incalculable—no hospital admissions in prior three years Low risk (<5) Intermediate risk (5–15) High risk (>15)	1,961 (8.2) 5,856 (24.5) 9,663 (40.4) 6,412 (26.8)	- 2,155 (15.9) 6,504 (48.0) 4,905 (36.2)	1,961 (19.0) 3,701 (35.8) 3,159 (30.6) 1,507 (14.6)
**Charlson Index before moving-in to care home** Incalculable—no hospital admissions in prior three years 0 comorbidities 1 comorbidity >1 comorbidities	1,961 (8.2) 6,761 (28.3) 7,279 (30.5) 7,891 (33.0)	- 2,880 (21.2) 4,969 (36.6) 5,715 (42.1)	1,961 (19.0) 3,881 (37.6) 2,310 (22.3) 2,176 (21.1)

IQR—interquartile range; SD—standard deviation

Requiring to receive nursing care after moving-in to the care home (*a proxy for complexity/dependency*) (adjusted Odds Ratio (aOR) 1.75 95% Confidence Interval (CI): 1.64–1.88), high frailty risk aOR 5.11 (95%CI: 4.60–5.68), increasing numbers of hospital admissions in the six months before moving-in ≥5 admissions aOR 3.64 (95%CI 2.60–5.09) versus 0–1 admissions and diagnoses of any fracture aOR 3.91 (95%CI: 3.41–4.47) or stroke aOR 8.42 (95%CI: 6.90–10.29) in the six months before moving-in were significant predictors of moving-in from hospital (Table 4). Discharge from in-patient psychiatry was also a highly significant predictor aOR 19.12 (95%CI: 16.26–22.48). Younger adults (aged <60 years) were less likely to move-in to a care home from hospital aOR 0.70 (95%CI 0.59–0.84) compared to those aged 80–89. Individuals with any diagnosis of dementia, or a hospital discharge with falls were less likely to move-in from hospital aOR 0.89 (95%CI 0.83–0.96) and aOR 0.88 (95%CI 0.80–0.96) after adjustment. Use of the HFRS as a measure of frailty in the adjusted model reduced the impact of individual factor, which were included in the HFRS (e.g. falls, incontinence).

**Table 4:** Regression model of factors associated with moving-in to a care home from hospital, compared to moving-in from the community

Factors	Moving-in from hospital (% of analysis cohort)	Unadjusted odds ratio (95% Confidence Interval)	Adjusted odds ratio (95% Confidence Interval)
**Age band moving-in to care home** <60 60–69 70–79 80–89 (*reference*) 90–99 >100	475 (40.7) 679 (61.7) 2,468 (57.7) 6,413 (57.8) 3,404 (56.5) 125 (53.6)	**0.50 (0.44–0.56)** **1.17 (1.03–1.33)** 1.00 (0.93–1.07)—0.95 (0.89–1.01) 0.84 (0.65–1.09)	**0.70 (0.59–0.84)** 1.16 (0.98–1.37) 0.92 (0.84–1.01)—1.05 (0.97–1.14) 1.10 (0.80–1.51)
**Sex** Female (*reference*) Male	8,568 (54.9) 4,996 (60.2)	- **1.24 (1.17–1.31)**	- **1.14 (1.06–1.22)**
**Funding** ^**a**^ NHS and Local Authority (*reference*) Private	9,281 (56.9) 4,276 (56.4)	- 0.97 (0.92–1.03)	- 1.04 (0.97–1.12)
**Receiving nursing care** No or missing (*reference*) Yes	3,664 (43.8) 9,900 (63.7)	- **2.25 (2.13–2.38)**	- **1.75 (1.64–1.88)**
**Dementia** (from SCHC (medically diagnosed) OR hospital discharge diagnosis in three years before moving-in to care home)	8,371 (57.3)	**1.06 (1.01–1.12)**	**0.89 (0.83–0.96)**
**Hospital discharge with diagnosis in three years before moving-in to care home** Cancer Chronic cardiovascular disease (including heart failure) Chronic respiratory disease Diabetes Falls Incontinence Mental and behavioural disorders due to use of alcohol Neurodegenerative disease (including Parkinson’s disease)	1,312 (74.0) 5,423 (69.9) 2,376 (70.8) 2,147 (70.1) 4,648 (70.7) 689 (75.1) 672 (67.8) 1,033 (70.0)	**2.29 (2.05–2.55)** **2.03 (1.87–2.20)** **2.03 (1.87–2.20)** **1.93 (1.78–2.10)** **2.28 (2.15–2.42)** **2.37 (2.04–2.76)** **1.64 (1.43–1.87)** **1.84 (1.64–2.07)**	**1.99 (1.75–2.26)** **1.31 (1.22–1.41)** **1.20 (1.09–1.32)** **1.23 (1.11–1.36)** **0.88 (0.80–0.96)** **1.32 (1.11–1.56)** 1.16 (0.98–1.38) **1.42 (1.24–1.62)**
**Hospital discharge in six months before moving-in to care home** Hospital discharge from in-patient psychiatry Hospital discharge with diagnosis of any fracture Hospital discharge with diagnosis of delirium Hospital discharge with diagnosis of stroke	2,032 (90.8) 2,157 (84.8) 1,656 (81.4) 1,133 (89.9)	**8.62 (7.45–9.96)** **4.84 (4.33–5.42)** **3.65 (3.25–4.09)** **7.32 (6.08–8.81)**	**19.12 (16.26–22.48)** **3.91 (3.41–4.47)** **1.84 (1.62–2.09)** **8.42 (6.90–10.29)**
**Number of hospitalisations in six months before moving-in to care home** 0–1 hospital admissions (*reference*) 2–4 hospital admissions ≥5 hospital admissions	7,037 (44.1) 6,242 (82.1) 285 (86.4)	- **5.83 (5.46–6.24)** **8.03 (5.86–11.02)**	- **2.97 (2.75–3.20)** **3.64 (2.60–5.09)**
**Number prescription drugs dispensed per month** 0 items dispensed 1–4 items dispensed (*reference*) 5–10 items dispensed >10 items dispensed	196 (39.3) 5,315 (56.4) 5,642 (56.8) 2,411 (59.6)	**0.50 (0.42–0.60)**—1.01 (0.96–1.07) **1.14 (1.06–1.23)**	1.05 (0.83–1.32)—**0.87 (0.81–0.94)** **0.76 (0.69–0.84)**
**Hospital Frailty Risk Score** [29] **before moving-in to care home** Low risk (<5) (*reference*)^b^ Intermediate risk (5–15) High risk (>15)	2,155 (27.6) 6,504 (67.3) 4,905 (76.5)	- **5.41 (5.07–5.77)** **8.55 (7.92–9.23)**	- **4.09 (3.76–4.44)** **5.11 (4.60–5.68)**

Bold results text denotes those that are statistically significant.

^a^Excludes 22 individuals where funding status is unknown.

^b^Low risk group includes 1,961 individuals with no hospital data to calculate Hospital Frailty Risk Score.

### Sensitivity analysis

Date of care home admission and hospital discharge matched exactly in 11,649 cases (85.9% of the hospital group in primary analysis). Results of the modelling using the exact match cohort as the ‘moving-in from hospital’ group are presented in Supplementary Table 2, with some age categories and funding status adjusting slightly and achieving statistical significance, but all other results consistent with the main analysis.

## Discussion

### Key findings in context

This national cross-sectoral data linkage analysis demonstrates systematic differences in the population moving-in to a care home directly from hospital compared to from community—in terms of care complexity, dependency, frailty and recent health events including fracture, stroke and significant mental illness. While such differences may have been anticipated by practitioners, they have not been previously described or quantified. The under-documentation of conditions prevalent in older adults reflects the limitation of using secondary care health data and the challenge of repurposing data collected in routine care for research. However, even with these limitations, the data provide useful insights into the complex health needs among those moving-in to care homes. The differences observed between the groups also provoke challenge around policy directives to reduce utilisation of long-term care and defer placement after a period of illness, if the two cohorts of individuals moving-in to care homes differ in their complexity and acuity of health needs.

Previous data linkage research involving UK care home residents typically lacks individual-level information from care home or social care records, often relying on information about the care home service (from publicly available data) and individual-level information from health records alone [37–39]. In comparison with international data, the current UK data infrastructure does not allow examination of constructs such as social vulnerability, which may contribute to the need to move-in to a care home [40]. Internationally, there is heterogeneity in long-term care provision with use of post-acute care facilities as a transition between acute care and home, associated with reduced use of long-term care placement, [41] and interest in using detailed data to aid in predicting need for care placement [42].

### Strengths & limitations

This work provides a national overview of a complex lived experience, making use of existing data resources without burdening individual residents or practitioners. The findings are inclusive of the care home population, not reliant on individual consent and the biases this can result-in. Thorough manual review work ensured only a small proportion of records were removed.

The findings support the potential of national data resources to improve understanding of the needs of those moving-in to care homes. However, this work has been time-consuming, with significant delays between project inception (autumn 2015) and analysis completion. Although some delays relate to ensuring understanding of the underlying data, many relate to the structural and practical challenges of undertaking data linkage research using new data sources. There have also been changes in local services and national policy around hospital discharge and care home assessment, which cannot be evaluated using these data from 2013 to 16. The COVID-19 pandemic has had a devastating impact on care homes in the UK, highlighting the gaps in national-level data about this population [43]. Initially care homes were used to create capacity within the NHS and ongoing service pressures are likely to affect pathways into care homes. Analysis of more contemporary data would be informative, now that our work has demonstrated feasibility of methods.

Submission to the SCHC is not mandatory, not all care homes submit individual-level resident data, thus not all individuals moving-in to care homes on a long-stay basis will be included. For our study period 74–81% of open homes contributed to the SCHC, including homes open only for respite/short stay purposes [44]. We do not have data about residents living in homes, which do not submit resident-level data to the SCHC.

The NHS data available in this study were limited to Scotland and thus hospital admissions and community prescribing data is only available for those resident in Scotland before moving-in to a care home in Scotland. It is possible that small numbers of individuals may have moved-in to care homes in Scotland from elsewhere and their comorbidities, frailty and prior diagnoses will not be captured.

The lack of nationally available primary care data results in an under-representation of the long-term conditions which individuals may experience [45] which do not result in hospitalisation and thus the true population morbidity is not adequately captured with secondary care data alone. Similarly, there was no comprehensive individual-level contemporaneous data on social care receipt to understand the inter-relationships, which may exist. Previous work using cross-sectional social care data has identified important associations between social care receipt and age, multimorbidity and area-based deprivation [46].

### Implications

Understanding pathways into care nationally provides useful information for service planning. That those moving-in from hospital are clinically distinct from those moving-in from the community is an important finding to target support for individuals and their families differently. This study focuses on those moving-in to care homes for long-stay purposes, further analysis is needed evaluating the role of temporary placements, including respite and intermediate care within care homes, to understand their role in long-term placement.

Identification of care home residency status within health data across the UK remains challenging, relying on algorithms and resulting in incomplete ascertainment [47, 48]. This continues to make the outcome measure of moving-in to a care home after hospitalisation, difficult to operationalise and monitor in everyday practice, despite the importance for individuals. NHS clinical systems should make use of Unique Property Reference Numbers and address-lookups to improve address recording and be sensitive to changes of information when an individual moves-in to a care home, even on a temporary basis [43].

There is increasing interest in using routinely collected large-scale data to advance ageing research [49]. While supportive of this aspiration, we would highlight the low prevalence of common conditions, including incontinence, depression and delirium superimposed on dementia (0 participants coded with this diagnosis in our cohort). While inclusion of primary care data may help, these examples highlight potential challenges in reliable identification of common conditions in older adults among routine data from practice, already recognised internationally [50]. As clinicians we must redouble our efforts to improve recording and data systems. Furthermore, we need to operationalise recording of key information such as formal and informal care, social networks and support and individual preferences within our electronic health records to ensure this information can be used for service evaluation and research with minimum additional manual effort.

Additional analysis exploring the outcomes and costs for these cohorts is planned as part of the UnPiCD study. However, the findings provoke questions around the different diagnostic trajectories (e.g. fractures, stroke) and hospital settings of care (particularly inpatient psychiatry) and the variations within the group moving-in to care homes from hospital. To fully understand the role of hospitalisation, it would be helpful to differentiate admission diagnoses from in-hospital adverse events. However, national hospital data do not consistently provide this level of detail, with diagnoses recorded at episode level at discharge without dates of occurrence.

The Office for Statistics Regulation has highlighted the imbalance in resources for social care and health statistics [51]. Although this study was based on a social care data resource, the imbalance is apparent with the bias favouring the availability and breadth of health data sources. There is a need to ensure social care data sources include meaningful measures which capture information important to those using services and this must be planned collaboratively [52]. While this research has made use of the unique data resource of the SCHC, our findings supports the case for review of its contents to ensure data collection is effective in summarising the complex needs of those living in Scotland’s care homes and better understanding of the services that support them.

## Conclusion

Linking social care data to health data is a promising approach. However, it requires targeted effort to operationalise meaningful information from both care home and health records facilitated by resource, infrastructure and governance. The clinical implications, that those moving-in to care homes from hospital have distinct needs from those moving-in from the community requires exploration with stakeholders on how these findings should influence pathways into care and the support provided to individuals and their families.

## Supplementary Material

aa-22-1373-File002_afac304
