# Supplementary material for: Understanding Pathways into Care homes using Data (UnPiCD study): a retrospective cohort study using national linked health and social care data
Source: Age Ageing. 2022 Dec 29;51(12):afac304. doi: 10.1093/ageing/afac304 (PMC9799248; doi:10.1093/ageing/afac304)
Supplement: aa-22-1373-File002_afac304 [file aa-22-1373-file002_afac304.docx]

**Understanding Pathways into Care homes using Data (UnPiCD study): a retrospective cohort study using national linked health and social care data**

**Supplementary Materials**

1. **Additional methods: Indexing and linkage methodology**
2. **Rules for manual review of cases where hospital date of discharge is after care home date of admission or date of discharge missing for one or more stay and the hospital stay has potential overlap with care home stay**
3. **Rules for manual review of cases where last episode location is not an NHS hospital location (non-H code)**
4. **Additional methods:** **Hospital episode linkage**
5. **ICD-10 codes for conditions in Scottish Morbidity Record Datasets**
6. **Additional results**

**Supplementary Table 1: Full cohort description of those moving-in to care homes from hospital and from the community**

**Supplementary Table 2: Sensitivity analysis results**

1. **Data availability**

**References**

1. **Additional methods**

**Indexing and linkage methodology**

The 10-digit unique identifier, the CHI number, is stored in the nationally-held CHI register (population spine), which includes all individuals in Scotland registered with a general practitioner.[1] The SCHC does not collect the resident’s CHI number, but includes other personal identifiers which can be used to assign individuals to their CHI number, in a process known as indexing. Indexing of the SCHC to the CHI Register was undertaken separately from the research team, by analysts from National Records of Scotland. The method and results of the linkage exercise are reported in detail elsewhere.[2] In summary, not all records were sufficiently complete in terms of available identifiers (name, date of birth and address) to enable them to be indexed to CHI. However, there were no biases around age, sex or deprivation status in the ability of records to be successfully indexed[2]. Records in the SCHC which could not be indexed to CHI were excluded.

Data were extracted by the team at the electronic Data Research and Innovation Service (eDRIS), part of Public Health Scotland (PHS). They received extracts from the Scottish Care Home Census (SCHC) of individuals with a record of care home admission in the study period. They then obtained extracts from National Records of Scotland (mortality data), the Scottish Morbidity Records and Prescribing Information System to obtain variables about the individuals identified in the SCHC. All records received an individual person-level identifier, so the same person could be found across all extracts if they had records in those data sources. All identifiable information was removed, including names, dates of birth and CHI numbers. Data extracts, by data source, were made available in the National Safe Haven for remote researcher access and analysis. The data were linked by the research team.

**2. Rules for manual review of cases where hospital date of discharge is after care home date of admission or date of discharge missing for one or more stay and the hospital stay has potential overlap with care home stay**

1. Check last location – if this is not a hospital location code [ending in H] – check previous episodes to confirm, moving-in to Care Home from hospital
   1. This includes NHS care homes in Scottish Morbidity Records (SMR) (see further below)
   2. This includes NHS hospices/private hospitals recorded in SMR
2. Timing of overlap – primary analysis definition allows care home admission to be -1 to -3 days before hospital discharge – if fits then classify as within_10
3. Review Scottish Care Home Census (SCHC) source of admission variable and hospital discharge coding (numerical variables)
   1. If SCHC states hospital or other/unknown and hospital discharge coding is to care home, institution, transfer (without a further hospital stay), temporary resident, usual place of residence institution, private residence – supported – essentially where SCHC and hospital coding matches – assign as discharged from hospital provided SCHC date of admission within -90 days of hospital discharge
4. Where date of hospital discharge is missing
   1. Check for subsequent hospital stays – may be clear the person was discharged (on unknown date) and later presents at hospital, discrete from stay before care home admission
   2. If no collateral information available – use SCHC source of admission variable
5. Remaining cases – SCHC and hospital coding do not match; SCHC date of admission >90 days before hospital discharge; death in hospital; overlapping episodes of hospital care covering hospital stay including missing dates of discharge – EXCLUDED as unable to reconcile information and allocate to group with confidence

**3. Rules for manual review of cases where last episode location is not an NHS hospital location (non-H code)**

1. Check sequence of episodes correctly identifies last episode of admission
2. If non-hospital location – check lookup table to identify if NHS hospice/private hospital – assign to moving-in from hospital group for primary analysis
3. If non-hospital location code – compare care home admission date with date of discharge within previous episodes – often the episode someone moves from the NHS hospital into the care home are included in the inpatient stay and the dates align. This can also identify if someone moves into another care home before moving to the care home where SCHC record is completed.

**4. Additional methods**

**Hospital episode linkage**

In SMR01/04/50 hospital admissions are composed of episodes of care (based on moving ward/department/hospital) and each episode of an inpatient stay has a location code. The episode data were linked into a single complete admission using the Continuous Inpatient Stay marker within the data, joining episodes ordered based on date of discharge. NHS Scotland data use common institution codes to classify hospitals, care homes, schools, prisons and other institutions in national datasets.[3] The last episode location before hospital discharge was checked against the institution code lookup for all those moving-in to a care home from hospital. This was done to ensure the last location was an NHS Hospital or Hospice and to identify NHS Care Home Locations which are included in national data. All NHS Hospital locations were further categorised into: Acute (hospitals with emergency departments), Acute – island (rural hospitals with emergency departments), Hospice or NHS Continuing Care (for individuals who require complex NHS-funded care in specialist facilities), Psychiatry and Rehabilitation & Community Hospitals.

**5. ICD-10 codes for conditions in Scottish Morbidity Record Datasets**

Anxiety disorders (F40, F41)

Arm fractures (S42, S52, S62)

Any fracture (S02, S12, S22, S32, S42, S52, S62, S72, S82, S92)

Bipolar affective disorder (F31)

Cancer (C00 to C97)

Chronic cardiovascular diseases including Heart Failure (I05-09, I25, I27, I34-7, I44-45, I48-9, I50)

Chronic kidney disease (N03, N04, N11, N18)

Chronic liver disease (K70, K72.1, K73, K74)

Chronic respiratory disease (J40-47, J60-70, J92, J96.1)

Confusion (disorientation, unspecified) (R41.0)

Delirium (F05)

Delirium superimposed on dementia (F05.1)

Dementia (F00, F01, F02, F03, G30, G31)

Depression (F32 & F33)

Diabetes (E10-E14)

Falls (W00-W19, R29.6)

Epilepsy (G40, G41)

Hip fracture (S72)

Incontinence (N39.3, N39.4, R15, R32)

Intracranial injury (S06)

Leg (non-hip) fractures (S82, S92)

Mental and behavioural disorders due to use of alcohol (F10)

Mental and behavioural disorders due to use of psychoactive substances (F11-19)

Neurodegenerative disease including Parkinson’s Disease (A81.0, F02.1, F02.2, G10-14, G20-26, G35-37, G70-73, G90-95)

Osteoporosis & fractures (M80)

Schizophrenia (F20) & Schizoaffective disorders (F25)

Stroke (I61, I63, I64)

Subarachnoid haemorrhage (I60)

Syncope and collapse (R55)

Vertebral and pelvic fracture (S12, S22, S32, M48.5, M49.5)

**6. Additional results**

**Cohort definition**

The analysis cohort was defined using the linked dataset. Individuals without SMR records were divided into those moving-in from the community or those moving-in from another care home. For individuals with SMR records, the definitions described above were applied. However, we identified individuals whose date of care home admission was before their date of hospital discharge (n=1,416) and those whose date of hospital discharge was missing for one episode within their hospital stay (n=26). A further 350 individuals had a non-hospital location code as their last location before moving-in to the care home. These 1,792 admissions (7.2% of those with SMR records) underwent additional manual review with rules applied, defined in Supplementary materials. Following this process 252 individuals (1.0% of those with SMR records) were removed from the analysis cohort as there was no way to reconcile the dates and data presented (e.g., date of care home admission >90 days before hospital discharge; deaths in hospital overlapping with period of care home admission; mismatch between SCHC source of admission and hospital discharge coding; death before recorded date of care home admission). These excluded records came from 203 care homes with a median of one (range one to three) record(s) per care home being excluded.

**Supplementary Table 1: Full cohort description of those moving-in to care homes from hospital and from the community**

|  | **Whole analysis**  **cohort**  N = 23,892 people (%) | **Moving-in**  **from hospital**  N = 13,564 people (%) | **Moving-in**  **from community**  N = 10,328 people (%) |
| --- | --- | --- | --- |
| **Census financial year moving-in to care home**  2013/14  2014/15  2015/16 | 8,282 (34.7)  8,085 (33.8)  7,524 (31.5) | 4,701 (34.7)  4,565 (33.6)  4,298 (31.7) | 3,582 (34.7)  3,520 (34.1)  3,226 (31.2) |
| Mean age moving-in to care home [SD]  Range of ages moving into care home | 82 years [11.96]  18-107 years | 83 years [10.45]  18-108 years | 82 years [13.65]  18-106 years |
| **Age band moving-in to care home**  <60 years  60-69  70-79  80-89  90-99  >100 years | 1,168 (4.9)  1,101 (4.6)  4,274 (17.9)  11,087 (46.4)  6,029 (25.2)  233 (1.0) | 475 (3.5)  679 (5.0)  2,468 (18.2)  6,413 (47.3)  3,404 (25.1)  125 (0.9) | 693 (6.7)  422 (4.1)  1,806 (17.5)  4,674 (45.3)  2,625 (25.4)  108 (1.0) |
| Male sex  Female sex | 8,299 (34.7)  15,593 (65.3) | 4,996 (36.8)  8,568 (63.2) | 3,303 (32.0)  7,025 (68.0) |
| White Ethnic Group  Other Ethnic Group  Ethnic Group not reported | 23,003 (96.3)  427 (1.8)  462 (1.9) | 13,071 (96.4)  235 (1.7)  258 (1.9) | 9.932 (96.2)  192 (1.9)  204 (1.9) |
| Mainly NHS funding  Mainly Local Authority funding  Mainly Private funding  Funding status missing | 612 (2.6)  15,670 (65.6)  7,588 (31.8)  22 (0.1) | 552 (4.1)  8,729 (64.3)  4,276 (31.5)  7 (0.1) | 60 (0.6)  6,941 (67.2)  3,312 (32.1)  15 (0.1) |
| Receiving personal care allowance  Not receiving personal care allowance  Not applicable^1^  Personal care allowance receipt missing | 6,597 (27.6)  999 (4.2)  16,255 (68.0)  41 (0.2) | 3,791 (27.9)  489 (3.6)  9,266 (68.3)  18 (0.1) | 2,806 (27.2)  510 (4.9)  6,989 (67.7)  23 (0.2) |
| Receiving nursing care allowance  Not receiving nursing care allowance  Not applicable^1^  Nursing care allowance receipt missing | 4,345 (18.2)  3,213 (13.5)  16,256 (68.0)  78 (0.3) | 2,803 (20.7)  1,453 (10.7)  9,266 (68.3)  42 (0.3) | 1,542 (14.9)  1,760 (17.0)  6,990 (67.7)  36 (0.4) |
| Receives nursing care  Not receiving nursing care  Nursing care receipt variable missing | 15,532 (65.0)  8,342 (34.9)  18 (0.1) | 9,900 (73.0)  3,654 (26.9)  10 (0.1) | 5,632 (54.5)  4,688 (45.4)  8 (0.1) |
|  | **Whole analysis**  **cohort** | **Moving-in**  **from hospital** | **Moving-in**  **from community** |
| **Conditions in Scottish Care Home Census**  Acquired brain injury  Dementia diagnosed  Dementia (not medically diagnosed)  Hearing impairment  Learning disability  Mental health problems excluding dementia  Other physical disability or chronic illness  Visual impairment  None of the Scottish Care Home Census conditions^2^ | 384 (1.6)  12,274 (51.4)  1,918 (8.0)  2,512 (10.5)  505 (2.1)  1,443 (6.0)  10,402 (43.5)  3,743 (15.7)  1,664 (7.0) | 269 (2.0)  6,715 (49.5)  1,078 (8.0)  1,413 (10.4)  165 (1.2)  907 (6.7)  6,583 (48.5)  2,089 (15.4)  929 (6.9) | 115 (1.1)  5,559 (53.8)  840 (8.1)  1,099 (10.6)  340 (3.3)  536 (5.2)  3,819 (37.0)  1,654 (16.0)  735 (7.1) |
| **Inpatient Hospital Diagnoses in three years before moving-in to care home**  Any fracture  Arm fractures  Cancer  Chronic cardiovascular disease (including heart failure)  Chronic kidney disease  Chronic liver disease  Chronic respiratory disease  Delirium  Delirium superimposed on dementia  Dementia  Depression  Diabetes  Epilepsy  Falls  Hip fracture  Incontinence  Intracranial injury  Leg (non-hip) fractures  Mental and behavioural disorders due to use of alcohol  Neurodegenerative conditions (including Parkinson’s disease)  Osteoporosis & fractures  Stroke  Subarachnoid haemorrhage  Syncope and collapse  Vertebral and pelvic fracture | 4,441 (18.6)  1,228 (5.1)  1,774 (7.4)  7,763 (32.5)  3,147 (13.2)  204 (0.9)  3,355 (14.0)  2,970 (12.4)  0  7,700 (32.2)  859 (3.6)  3,062 (12.8)  582 (2.4)  6,570 (27.5)  2,431 (10.2)  917 (3.8)  280 (1.2)  400 (1.7)  991 (4.2)  1,475 (6.2)  267 (1.1)  1,998 (8.4)  61 (0.3)  2,028 (8.5)  983 (4.1) | 3,238 (23.9)  873 (6.4)  1,312 (9.7)  5,423 (40.0)  2,245 (16.6)  143 (1.1)  2,376 (17.5)  2,235 (16.5)  0  5,319 (39.2)  621 (4.6)  2,147 (15.8)  383 (2.8)  4,648 (34.3)  1,819 (13.4)  689 (5.1)  220 (1.6)  277 (2.0)  672 (4.9)  1,033 (7.6)  191 (1.4)  1,592 (11.7)  49 (0.4)  1,356 (10.0)  730 (5.4) | 1,203 (11.6)  355 (3.4)  462 (4.5)  2,340 (22.7)  902 (8.7)  61 (0.6)  979 (9.5)  735 (7.1)  0  2,381 (23.1)  238 (2.3)  915 (8.9)  199 (1.9)  1,922 (18.6)  612 (5.9)  228 (2.2)  60 (0.6)  123 (1.2)  319 (3.1)  442 (4.3)  76 (0.7)  406 (3.9)  12 (0.1)  672 (6.5)  253 (2.5) |
|  | **Whole analysis**  **cohort** | **Moving-in**  **from hospital** | **Moving-in**  **from community** |
| **Inpatient Psychiatry Diagnoses in three years before moving-in to care home**  Anxiety disorders  Bipolar affective disorder  Delirium  Delirium superimposed on dementia  Dementia  Depression  Mental and behavioural disorders due to use of alcohol  Mental and behavioural disorders due to use of psychoactive substances  Schizophrenia & Schizoaffective disorders | 116 (0.5)  102 (0.4)  86 (0.4)  0  1,786 (7.5)  292 (1.2)  285 (1.2)  57 (0.2)  241 (1.0) | 100 (0.7)  73 (0.5)  64 (0.5)  0  1,570 (11.6)  230 (1.7)  202 (1.5)  31 (0.2)  185 (1.4) | 16 (0.2)  29 (0.3)  22 (0.2)  0  216 (2.1)  62 (0.6)  83 (0.8)  26 (0.3)  56 (0.5) |
| **Hospital use and significant events in six months before moving-in to care home**  Mean number of hospital admissions per person [SD]  Median cumulative length of hospital stay per person [IQR]  Hospital discharge from in-patient psychiatry  Hospital discharge with diagnosis of cancer  Hospital discharge with diagnosis of dementia  Hospital discharge with diagnosis of delirium  Hospital discharge with diagnosis of fracture  Hospital discharge with diagnosis of stroke | 1.2 admissions [1.15]  70 days [34-116]  2,239 (9.4)  1,247 (5.2)  6,740 (28.2)  2,035 (8.5)  2,545 (10.7)  1,260 (9.4) | 1.7 admissions [1.06]  84 days [51-131]  2,032 (15.0)  1,020 (7.5)  5,436 (40.1)  1,656 (12.2)  2,157 (15.9)  1,133 (8.4) | 0.6 admissions [0.92]  21 days [8-50]  207 (2.0)  227 (2.2)  1,304 (12.6)  379 (3.7)  388 (3.8)  127 (1.2) |
| **Community medication use in three years before moving-in to care home**  Mean frequency of prescriptions [SD]  Range frequency of prescriptions  Mean dispensed items/month [SD]  Range dispensed items per month | 29.2 days [33.36]  0 to 1,076 days  6.5 items [5.18]  0 to 84 items | 29.2 days [31.85]  0 to 966 days  6.6 items [5.24]  0 to 84 items | 29.3 days [35.24]  0 to 1,076 days  6.3 items [5.10]  0 to 78 items |
| **Hospital Frailty Risk Score** **before moving-in to care home**  Incalculable – no hospital admissions in prior three years  Low Risk (<5)  Intermediate Risk (5-15)  High Risk (>15) | 1,961 (8.2)  5,856 (24.5)  9,663 (40.4)  6,412 (26.8) | -  2,155 (15.9)  6,504 (48.0)  4,905 (36.2) | 1,961 (19.0)  3,701 (35.8)  3,159 (30.6)  1,507 (14.6) |
| **Charlson Index** **before moving-in to care home**  Incalculable – no hospital admissions in prior three years  0 comorbidities  1 comorbidity  >1 comorbidities | 1,961 (8.2)  6,761 (28.3)  7,279 (30.5)  7,891 (33.0) | -  2,880 (21.2)  4,969 (36.6)  5,715 (42.1) | 1,961 (19.0)  3,881 (37.6)  2,310 (22.3)  2,176 (21.1) |

Footnotes 1. Variable on personal and nursing care only collected for those whose main funding source is private

2. Scottish Care Home Census also collects alcohol-related problems, drugs-related problems which were not requested as variables for analysis in this project – thus this category applies to those without any of the conditions specified in the Census

**Supplementary Table 2: Sensitivity analysis results** *(Moving-in from hospital group includes only those whose hospital date of discharge is an exact match to the date of moving-in to the care home, excluding manual matching)*

| **Factors** | **Moving-in from hospital**  **(% of analysis cohort)** | **Adjusted Odds Ratio**  **(95% Confidence Interval)** | **Comparison with main**  **analysis cohort results** |
| --- | --- | --- | --- |
| **Age band moving-in to care home**  <60  60-69  70-79  80-89 (*reference*)  90-99  >100 | 475 (40.7)  679 (61.7)  2,468 (57.7)  6,413 (57.8)  3,404 (56.5)  125 (53.6) | **0.63 (0.52-0.76)**  1.06 (0.89-1.27)  **0.88 (0.80-0.97)**  -  **1.09 (1.01-1.18)**  1.17 (0.84-1.62) | Consistent  Consistent  SA becomes statistically significant  -  SA becomes statistically significant  Consistent |
| **Sex**  Female (*reference*)  Male | 8,568 (54.9)  4,996 (60.2) | -  **1.13 (1.05-1.22)** | -  Consistent |
| **Funding^1^**  NHS and Local Authority (*reference*)  Private | 9,281 (57.0)  4,276 (56.4) | -  **1.08 (1.01-1.16)** | -  SA becomes statistically significant |
| **Receiving nursing care**  No or missing (*reference*)  Yes | 3,664 (39.4)  9,900 (67.8) | -  **1.74 (1.63-1.87)** | -  Consistent |
| **Dementia** (from SCHC (medically diagnosed) OR hospital discharge diagnosis in three years before moving-in to care home) | 8,371 (57.3) | **0.89 (0.83-0.96)** | Consistent |
| **Hospital discharge with diagnosis in three years before moving-in to care home**  Cancer  Chronic cardiovascular disease (including heart failure)  Chronic respiratory disease  Diabetes  Falls  Incontinence  Mental and behavioural disorders due to use of alcohol Neurodegenerative disease (including Parkinson’s disease) | 1,312 (74.0)  5,423 (69.9)  2,376 (70.8)  2,147 (70.1)  4,648 (70.7)  689 (75.1)  672 (67.8)  1,033 (70.0) | **1.92 (1.69-2.20)**  **1.30 (1.21-1.40)**  **1.19 (1.08-1.31)**  **1.26 (1.14-1.40)**  **0.85 (0.78-0.93)**  **1.35 (1.14-1.61)**  1.19 (0.99-1.42)  **1.46 (1.27-1.68)** | Consistent  Consistent  Consistent  Consistent  Consistent  Consistent  Consistent  Consistent |
| **Hospital discharge in six months before moving-in to care home**  Hospital discharge from in-patient psychiatry  Hospital discharge with diagnosis of any fracture  Hospital discharge with diagnosis of delirium  Hospital discharge with diagnosis of stroke | 2,032 (90.8)  2,157 (84.8)  1,656 (81.4)  1,133 (89.9) | **22.68 (19.18-26.69)**  **4.06 (3.54-4.65)**  **1.90 (1.67-2.16)**  **8.95 (7.30-10.97)** | Consistent  Consistent  Consistent  Consistent |
| **Factors (continued)** | **Moving-in from hospital**  **(% of analysis cohort)** | **Adjusted Odds Ratio**  **(95% Confidence Interval)** | **Comparison with main**  **analysis cohort results** |
| **Number of hospitalisations in six months before moving-in to care home**  0-1 hospital admissions (*reference*)  2-4 hospital admissions  ≥5 hospital admissions | 7,037 (44.1)  6,242 (82.1)  285 (86.4) | -  **2.83 (2.61-3.06)**  **3.48 (2.47-4.91)** | -  Consistent  Consistent |
| **Number prescription drugs dispensed per month**  0 items dispensed  1-4 items dispensed (*reference*)  5-10 items dispensed  >10 items dispensed | 196 (39.3)  5,315 (56.4)  5,642 (56.8)  2,411 (59.6) | 1.03 (0.80-1.33)  -  **0.86 (0.80-0.93)**  **0.76 (0.69-0.84)** | Consistent  -  Consistent  Consistent |
| **Hospital Frailty Risk Score before moving-in to care home**  Low risk (<5) (*reference*)^2^  Intermediate risk (5-15)  High risk (>15) | 2,155 (27.6)  6,504 (67.3)  4,905 (76.5) | -  **4.32 (3.96-4.72)**  **5.53 (4.95-6.17)** | -  Consistent  Consistent |

Footnotes

SA: sensitivity analysis result

1. Excludes 22 individuals where funding status is unknown

2. Low risk group includes 1,961 individuals with no hospital data to calculate Hospital Frailty Risk Score

**7. Data availability**

The linked data for this project (1516-0438) are held in the NHS Scotland National Safe Haven managed by the electronic Data Research and Innovation Service (eDRIS), part of Public Health Scotland. Application to the Public Benefit and Privacy Panel for approval is required to request access to the data used here. The data controllers for the data sources are: Public Health Scotland, Scottish Government and National Records of Scotland. Access to programming code would be reviewed on an individual basis by contacting the research team and is subject to Disclosure Control by the eDRIS team.

**References**

1. Womersley J. The public health uses of the Scottish Community Health Index (CHI). Journal of public health medicine. 1996;18(4):465-72.

2. Henderson D, Burton J, Lynch E, Rintoul J, Clark D, Bailey N. Data Resource Profile The Scottish Social Care Survey (SCS) and the Scottish Care Home Census (SCHC). International Journal of Population Data Science. 2019;4:24.

3. National Records of Scotland. Code-lists Used in Vital Event Statistics: Institutions. 2022 [cited 2021 18th October]; Available from: <https://www.nrscotland.gov.uk/files//statistics/vital-events/institution-codes-october-2021.xlsx>
